# Deep-Learning-Enabled Computer-Aided Diagnosis in the Classification of Pancreatic Cystic Lesions on Confocal Laser Endomicroscopy †

**DOI:** 10.3390/diagnostics13071289

**Published:** 2023-03-29

**Authors:** Tsung-Chun Lee, Clara Lavita Angelina, Pradermchai Kongkam, Hsiu-Po Wang, Rungsun Rerknimitr, Ming-Lun Han, Hsuan-Ting Chang

**Affiliations:** 1 Division of Gastroenterology and Hepatology, Department of Internal Medicine, Shuang Ho Hospital, Taipei Medical University, New Taipei City 23561, Taiwan; johnlee0212@gmail.com; 2 Department of Internal Medicine, School of Medicine, College of Medicine, TMU Research Center for Digestive Medicine, Taipei Medical University, Taipei 11031, Taiwan; 3 Department of Electrical Engineering, National Yunlin University of Science and Technology, Yunlin 64002, Taiwan; claralavita52@gmail.com; 4 Excellent Center for Gastrointestinal Endoscopy and Division of Gastroenterology, King Chulalongkorn Memorial Hospital, Chulalongkorn University, Bangkok 10330, Thailand; kongkam@hotmail.com (P.K.);; 5 Pancreas Research Unit, Division of Hospital and Ambulatory Medicine, Department of Medicine, Faculty of Medicine, Chulalongkorn University, Bangkok 10330, Thailand; 6 Division of Gastroenterology and Hepatology, Department of Internal Medicine, College of Medicine, National Taiwan University Hospital, National Taiwan University, Taipei 10002, Taiwan; wanghp@ntu.edu.tw; 7 Department of Integrated Diagnostics and Therapeutics, National Taiwan University Hospital, Taipei 10002, Taiwan; minglun@ms18.hinet.net

**Keywords:** deep learning, pancreatic cystic lesions, VGG19, U-Net, confocal laser endomicroscopy, computer-aided diagnosis, endoscopic ultrasound

## Abstract

Accurate classification of pancreatic cystic lesions (PCLs) is important to facilitate proper treatment and to improve patient outcomes. We utilized the convolutional neural network (CNN) of VGG19 to develop a computer-aided diagnosis (CAD) system in the classification of subtypes of PCLs in endoscopic ultrasound-guided needle-based confocal laser endomicroscopy (nCLE). From a retrospectively collected 22,424 nCLE video frames (50 videos) as the training/validation set and 11,047 nCLE video frames (18 videos) as the test set, we developed and compared the diagnostic performance of three CNNs with distinct methods of designating the region of interest. The diagnostic accuracy for subtypes of PCLs by CNNs with manual, maximal rectangular, and U-Net algorithm-designated ROIs was 100%, 38.9%, and 66.7% on a per-video basis and 88.99%, 73.94%, and 76.12% on a per-frame basis, respectively. Our per-frame analysis suggested differential levels of diagnostic accuracy among the five subtypes of PCLs, where non-mucinous PCLs (serous cystic neoplasm: 93.11%, cystic neuroendocrine tumor: 84.31%, and pseudocyst: 98%) had higher diagnostic accuracy than mucinous PCLs (intraductal papillary mucinous neoplasm: 84.43% and mucinous cystic neoplasm: 86.1%). Our CNN demonstrated superior specificity compared to the state-of-the-art for the classification of mucinous PCLs (IPMN and MCN), with high specificity (94.3% and 92.8%, respectively) but low sensitivity (46% and 45.2%, respectively). This suggests the complimentary role of CNN-enabled CAD systems, especially for clinically suspected mucinous PCLs.

## 1. Introduction

Pancreatic cystic lesions (PCLs) have a prevalence rate of 2.4% to 24.3% in the asymptomatic adult population [1,2]. Common PCLs consist of five main subtypes, and each presents different disease courses and aggressiveness: (1) intraductal papillary mucinous neoplasm (IPMN), (2) mucinous cystic neoplasm (MCN), (3) serous cystic neoplasm (SCN), (4) cystic neuroendocrine tumor (NET), and (5) pseudocysts [3]. Differentiation among subtypes of PCLs is critical, as mucinous PLCs have higher cancer risk. Advanced neoplasia was reported in 100% of the main ductal-type of IPMN, in 39% of resected MCN, in 30% of branch-type IPMN, and in 10% of resected NET [4]. Subtyping will impact the clinical decision on the surgical management and non-surgical surveillance, as SCN and pseudocysts have a low cancer risk, for which costly surveillance could be avoided. Abdominal ultrasound, computed tomography, magnetic resonance imaging, and endoscopic ultrasound (EUS) are utilized to evaluate PCLs [5]. However, accurate preoperative diagnosis of subtypes of PCLs poses practical challenges to clinicians.

Confocal laser endomicroscopy (CLE), a novel endoscopy technology with real-time 1,000-fold magnification, enables in vivo optical pathology for various diseases in multiple organ systems [6]. CLE enables the direct visualization of elastin fibers of bronchus and structural changes of alveoli in bronchial asthma and interstitial lung disease, respectively [7]. In addition, CLE could enable the direct visualization of lung cancer cells and the potential of monitoring post-chemotherapy responses by direct observing the apoptosis of cancer cells.

EUS-guided needle-based confocal laser endomicroscopy (nCLE) enables in vivo optical pathology to examine PCLs [6]. In a systematic review and international Delphi report with fifteen nCLE experts, twelve clinical studies were reviewed. Characteristic nCLE features enabled differentiation of mucinous versus non-mucinous PCLs with an accuracy of 71–93% and serous cystadenoma versus non-serous PCLs with an accuracy of 87–99% [8].

In addition to differentiation of subtypes of PCLs, nCLE was also shown to provide risk stratification for malignant potential of IPMNs. Krishna et al. investigated characteristic findings of nCLE on 26 IPMNs (including 16 cases of high-grade dysplasia and cancers) and found that the quantification of papillary epithelial width and darkness on nCLE had high accuracy to identify cases of high-grade dysplasia and cancers [9].

Krishna et al. investigated 29 nCLE videos on PCLs with six expert endosonographers and showed that the diagnostic accuracy and interobserver agreement (IOA) were 95%, k = 0.81 for mucinous PCLs, and 98%, k = 0.83 for SCN, respectively [10]. Machicado et al. utilized 76 nCLE videos from three prospective studies and invited 13 expert endosonographers to test the diagnostic accuracy and IOA [11]. The diagnostic accuracy for IPMN, MCN, SCN, cystic-NET, and pseudocyst revealed 86%, 84%, 98%, 96%, and 96%, and IOA k = 0.72, 0.47, 0.85, 0.73, and 0.57, respectively. Nevertheless, for non-experts, real-time interpretation of nCLE can be time-consuming and requires specialized operator training [12].

Traditional machine learning methods encompass linear discriminants, Bayesian networks, random forest, and support vector machine, while modern machine learning methods consist of artificial neural networks and convoluted neural network (CNN) [13]. Applications of deep learning CNN technologies in endoscopy have created an exciting new era of computer-aided diagnosis (CAD) in endoscopy [14]. For example, Rashid et al. combined radiomics of feature extraction and CNN of featureless methods to improve the detection accuracy of breast lesions [15], and appropriate optimization methods have been shown to further enhance the results of CNN [16].

Two recent studies utilizing CNN-enabled CAD systems tried to address issues related to nCLE, such as long learning curve, low kappa value in readings, and time consumed by endoscopists [17,18].

For CAD systems on nCLE videos, designation of ROIs in each frame of the video constitutes a practical problem to solve in the first place, especially when the 0.85 mm miniproble examines inside the PCLs.

We compared three different region of interest (ROI) designations and used VGG19 as the classifier: (1) CNN1: manually designated ROIs, (2) CNN2: maximal rectangular ROIs, and (3) CNN3: U-Net algorithm-designated ROIs.

In this study, we aimed to develop CAD classification system to differentiate subtypes of PCLs on nCLE and to investigate three different ways of designating ROI. Our work will contribute to solving the daily dilemma for endoscopists in classifying subtypes of PCLs on nCLE, which is the very first step in detecting mucinous PCLs of high malignant potential.

## 2. Materials and Methods

### 2.1. Patients

We retrospectively collected 68 de-identified nCLE videos (IPMN: 31, MCN: 10, SCN: 18, NET: 8, and pseudocyst: 12) on PCLs with histologically and/or clinically confirmed diagnosis from King Chulalongkorn Memorial Hospital, Bangkok, Thailand. All the nCLE procedures were performed by an experienced endoscopist (P.K.) with an AQ-Flex nCLE mini-probe (Cellvizio, Mauna Kea Technologies, Paris, France) after intravenous fluorescein. We also collected IPMN, MCN, and pseudocyst videos from publicly available sources [10,19]. The research protocol was approved by the Institutional Review Board of the Faculty of Medicine, Chulalongkorn University (960/64, Dec 2021; 0127/66, Feb 2023), and the study was conducted in accordance with the Declaration of Helsinki.

For training and validation set images, we randomly selected nCLE videos within each subtype for a total of 50 videos for training and validation sets. Among the total of 21,937 images, we used a 70–30% ratio to randomly divide into training sets and validation sets per subtype of PCLs. The training set consisted of the following: IPMN (26 videos, 3122 images), MCN (5 videos, 1249 images), SCN (9 videos, 3239 images), NET (4 videos, 4220 images), and pseudocyst (6 videos, 3526 images), respectively. The validation set consisted of 6581 images.

The remaining 18 nCLE videos were used for the test set, including five IPMN videos (collectively 2537 images), three MCN videos (1557 images), four SCN videos (3693 images), four NET videos (2482 images), and two pseudocyst videos (778 images).

### 2.2. CAD System Overview

**Figure 1:**
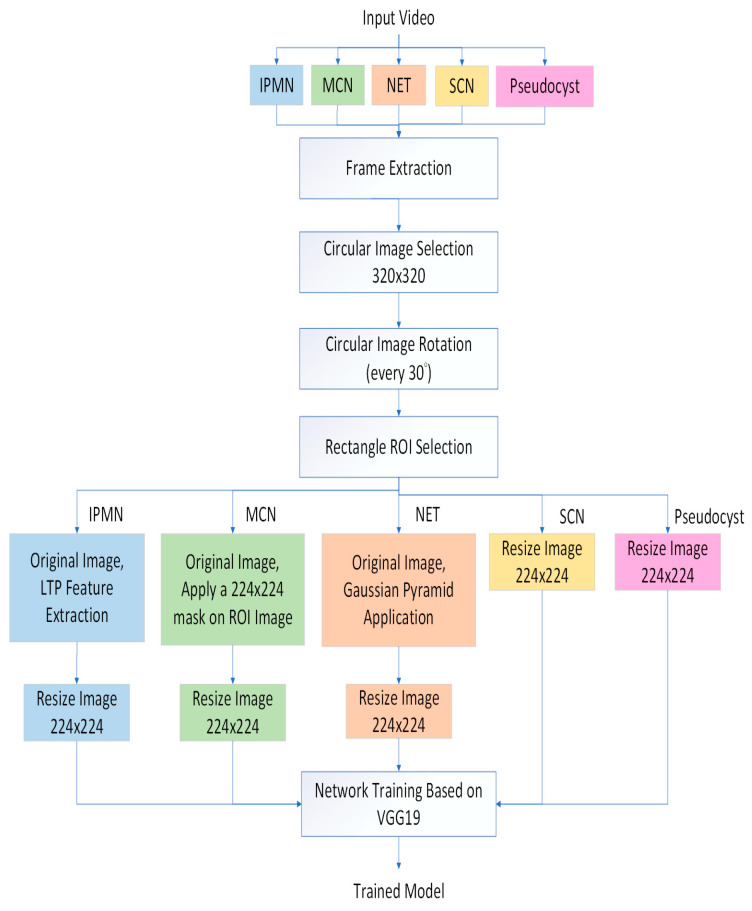
Training stage overview.

**Figure 2:**
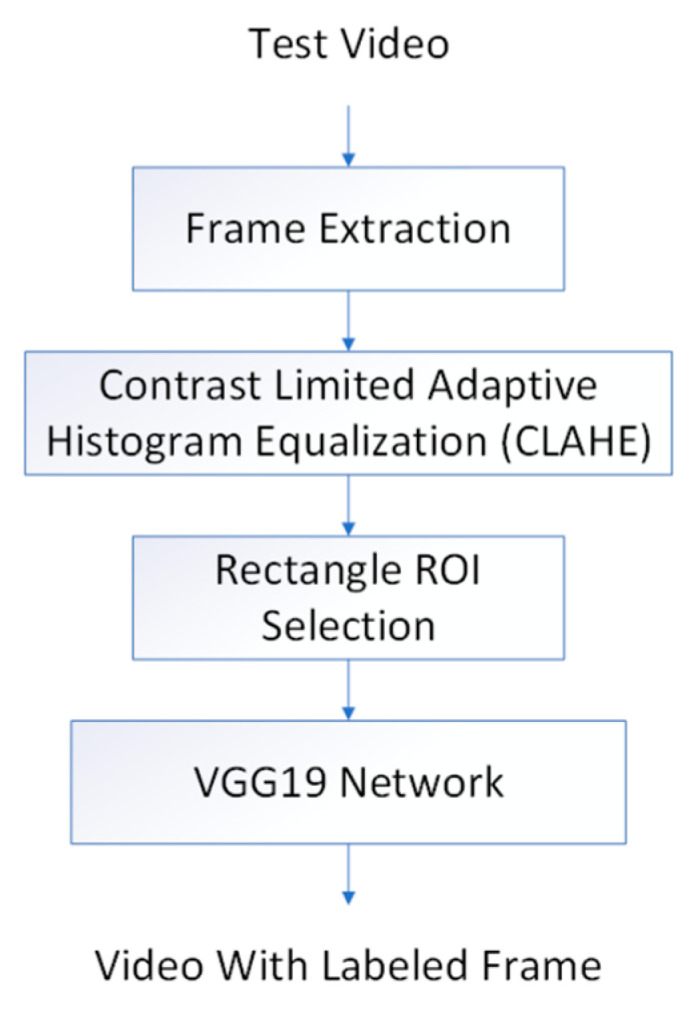
Test stage overview.

The proposed method was divided into the training and test stages shown in Figure 1 and Figure 2, respectively. In the training stage (Figure 1), we firstly performed image preprocessing (Gaussian pyramid application and local ternary pattern feature extraction) [20] (Supplementary Materials) and data augmentation. For regions of interest (ROI), we attempted three methods: (1) manual designation of ROIs, (2) maximal-sized rectangular ROIs, and (3) automatic designation of ROIs by another deep learning algorithm, U-Net. Finally, we utilized the deep learning algorithm, VGG19, as our classifier of subtypes.

In the test stage (Figure 2), we applied the contrast-limited adaptive histogram equalization (CLAHE) preprocessing on the test video frames to enhance image contrast [21]. The trained VGG19 algorithm was used to classify the PCL subtype frame by frame of the test set videos. The final classification of PCL subtype for the whole test video was then determined by the most frequent subtype.

### 2.3. CNN Architecture of VGG19

In this study, we utilized the VGG19 deep learning network of 19 layers to classify PCLs. VGG19 was proposed by the Visual Geometry Group (VGG) at Oxford University [22]. The VGG19 network architecture consists of 19 convolution weight layers with 3 × 3 kernel sizes, 5 Maxpool layers with 2 × 2 pool sizes, and a final output layer with the Softmax activation function. The reason for using Softmax is its ability to execute multiclass-class classification. In the training stage, we used the Adam optimizer with 100 epochs, and input images were fixed at sizes of 224 × 224 pixels. The sensitivity analysis of para-meters in the algorithms was described in the Supplementary Materials. 

### 2.4. Designation of ROIs: (1) Manually Designated ROI, (2) Maximal Rectangular ROI, and (3) U-Net Algorithm-Designated ROI

**Figure 3:**
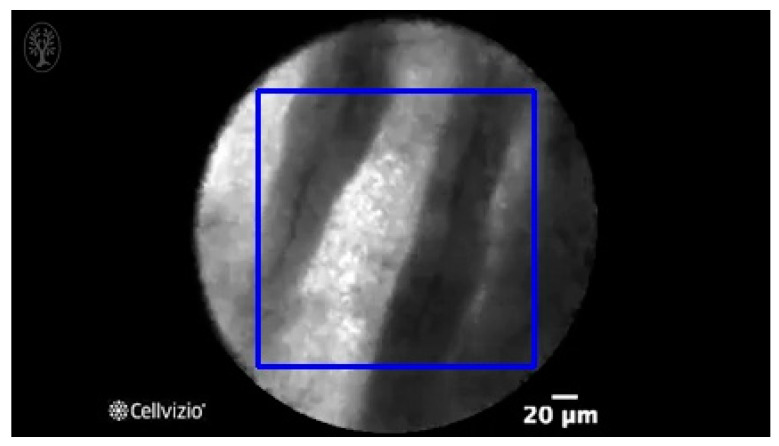
An exemplary view of maximal rectangular ROI (blue) on nCLE video frame of IPMN, which shows papillary epithelia and central vascular cores.

Manual designation of ROIs in CNN1 was performed by selection of the most prominent image features in each frame (as shown in Table 1), while the maximal size of rectangle in each frame was designated as the ROI in CNN2 (as shown in Figure 3).

In CNN3, we trained another deep learning algorithm, U-Net, to automatically segment the ROI in each frame (as shown in Table 2). 

**Figure 4:**
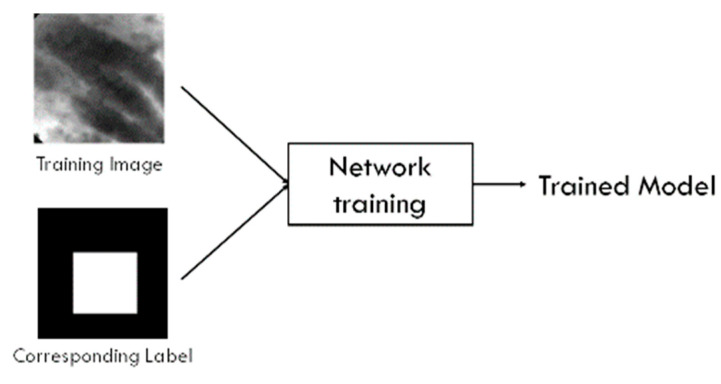
U-Net training system overview.

While VGG is designed for classification tasks (output: categories) but not for segmentation tasks (output: ROI within a given image), U-Net has been utilized in the tasks of segmentation in medical images with good performance [23,24,25,26,27,28]. We created a dataset with all PCLs and the corresponding label (ground truth) that stated the difference between image features and the image background. The U-Net was then trained using 1200 training images and their PCLs labels. Figure 4 illustrates the U-Net model training process. The learning rate was 0.001, and the learning rate optimizer was adaptive moment estimation (Adam). For the loss function, we used binary cross-entropy. The training batch size was set at 8 and the epoch 100.

**Figure 5:**
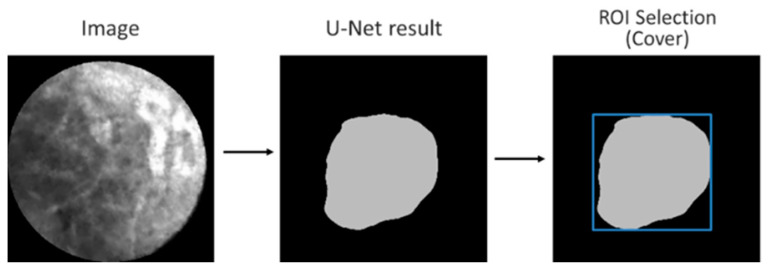
U-Net ROI designation process.

Figure 5 illustrates the ROI designation process for a given test image. First, the output of the trained U-Net was an irregular boundary. Then, the minimally-sized square (shown in blue) covering the entire region automatically formed the boundaries of the ROI. Thus, the ROI could be automatically designated.

### 2.5. Data Augmentation

**Figure 6:**
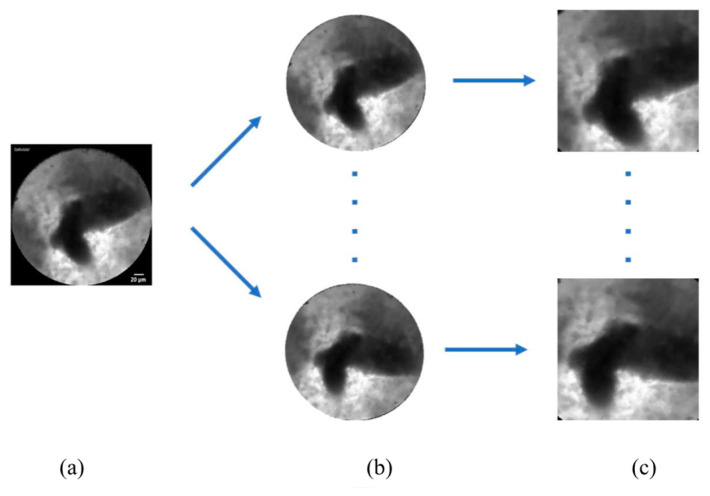
Data augmentation by rotation every 30 degrees to generate 12 different angles of images. (**a**) Original frame; (**b**) rotated circular image every 30 degrees; (**c**) square ROI from rotated images.

We performed data augmentation to expand the numbers of images for training (Figure 6). Because the original nCLE video images were circular, we rotated the original images every 30 degrees to obtain 12 different angles of images.

### 2.6. Hardware and Software Specifications

The system was operating in a PC with Windows 10 Professional Edition (64-bit), with an Intel CPU Core i7-9700 at 3.2 GHz, 64 GB-DDR4 memory, and NVIDIA GeForce RTX2080Ti graphics card. The software platform was Visual Studio 2017 with OpenCV library, and the programming language was Python 3.6.

### 2.7. Statistics

Descriptive data are reported as proportions for categorical variables and means +/− SD for continuous variables. For statistical analysis, the chi-square test was performed for categorical variables and *t*-test for continuous variables. Statistical significance was defined as *p* < 0.05. The classification performances of deep learning algorithms with three different ROI selections were determined against the ground truth (histologically and/or clinically confirmed diagnosis). For frame-by-frame basis, the sensitivity, specificity, positive predictive value, negative predictive value, and accuracy were calculated per frame and are shown as percentages with 95% confidence interval (CI). All analyses were performed using SAS software version 9.4 (Cary, NC, USA).

## 3. Results

After preprocessing with Gaussian pyramid, LTP extraction, and CLAHE, we utilized the CNN VGG19 as the classifier for five subtypes of PCLs in the 18 test nCLE videos.

In the training stage, we spent six hours training all of images using the VGG19 network: about 216 s per epoch. We also calculated the computation time in processing test nCLE videos. The average time for processing one frame is 0.05 s/frame, as shown in Table 3.

We evaluated the performance of our three CAD systems of CNN1 (manually designated ROIs), CNN2 (maximal rectangular ROIs), and CNN3 (U-Net algorithm-designated ROIs) on the per-video and per-frame bases.

### 3.1. Performance of Three CNNs on the Per-Video Basis

Table 1 and Table 2, Figure 3, show the exemplary views of designated ROIs in CNN1, CNN2, and CNN3, respectively. Table 4, Table 5 and Table 6 reveal the results of subtypes on a per-video basis in CNN1, CNN2, and CNN3, respectively. Manually designated ROIs in CNN1 achieved the highest accuracy rate of 100% (18/18 videos), followed by U-Net algorithm-designated ROIs in CNN3 of 66.7% (12/18 videos). In contrast, maximal rectangular ROIs in CNN2 had the lowest accuracy rate of 38.9% (7/18 videos).

### 3.2. Performance of Three CNNs on the Per-Fame Basis

Table 7 summarizes the performance of three CNNs on the per-frame basis in relation to five subtypes of PCLs. CNN1 achieved the highest average accuracy of 88.99%, followed by CNN3 at 76.12% and CNN2 at 73.94%.

Among the five subtypes, pseudocyst had the highest sensitivity (94.22%, 95% CI 92.34–95.75%), specificity (98.29%, 95% CI 95.75–98.02%), and accuracy (98%, 95% CI 97.72–98.25%) in CNN1.

MCN, among the five subtypes, had the lowest sensitivity (45.22% in CNN1, 7.11% in CNN2, and 8.54% in CNN3) but consistently high specificity (92.81% in CNN1, 99.4% in CNN2, and 95.97% in CNN3).

IPMN, as the main research focus of nCLE in published studies, exhibited consistently high specificity (94.31%, 96.35%, and 87.39%) and good accuracy (83.43%, 80.68%, and 76.62%), but relatively low sensitivity (45.95%, 28.14%, and 40.48%) in CNN1-3, respectively.

Our results also demonstrated the volatile performance of classifying SCN and NET across three different methods of ROI designations, which suggests the importance of ROI designation in diagnosing SCN and NET by nCLE.

## 4. Discussion

To the best of our knowledge, our study is the first report utilizing deep learning CAD systems to classify the subtypes of PCLs on EUS-guided nCLE video frames. Our results demonstrate the feasibility of applying novel CNN technologies to classify common subtypes of PCLs on a per-video and per-frame basis.

Our exploration of three different methods of designating ROIs on a rapidly changing frame-by-frame nCLE video originated from practical clinical considerations. Although manual selection of ROIs might achieve high accuracy owing to selection bias, our results showed a promising automatic ROI designation by another deep learning algorithm, namely U-Net. In our results, CNN3 delivered a 66.7% accuracy rate per video and 76.12% rate per frame. Such automatic ROI designation could further assist the subtype classification of nCLE on PCLs, especially for non-expert endoscopists.

There have been attempts to adopt CNN in nCLE images for PCLs (Table 8). Kuwahara et al. utilized a CNN algorithm of ResNet50 to analyze 3,970 still images of linear EUS on pathology-confirmed IPMNs. The mean AI value (output value of the TensorFlow algorithm) was shown to be higher in malignant IPMNs than that in non-malignant IPMNs (0.808 vs 0.104, *p* < 0.001), which provided a tool for risk stratification of IPMNs [17]. Machicado et al. utilized 15,027 image frames from 35 IPMN nCLE videos and applied the CNN algorithm of VGG16 [18]. They developed two CAD models: a guided (epithelial thickness and darkness in papillary structures) segmentation-based model (SBM) targeting papillary epithelial thickness and darkness and a holistic-based model (HBM), in which the model automatically extracted nCLE features for IPMN malignant risk stratification. The study showed promising results when compared with clinical diagnosis guidelines: diagnostic accuracy SBM: 82.9%; HBM: 85.7%; and guidelines 68.6% and 74.3%, respectively. Of special note, Machicado et. al addressed the issue of automatic ROI designation in attempts using a mask region-based CNN in their segmentation-based model and another VGG-16 network in their holistic-based model. In this regard, we tried an additional CNN, i.e., U-Net, to automatically segment the ROIs in CNN3, which performed less well than CNN1 (manually designated ROIs).

Kurita et al. utilized basic deep learning neural network on the analysis of pancreatic cystic fluid in the differential diagnosis of mucinous PCLs [29]. Meanwhile, Liang et al. utilized support vector machine on the classification of mucinous versus non-mucinous PCLs. Both studies only focused on the differentiation of mucinous PCLs [30].

Our study differed from the aforementioned studies in that we tried to solve the initial step of subtyping of nCLE on PCLs for practicing EUS endoscopists, for whom training to master skills has been a challenge [31]. CAD might shorten the learning curve and have the potential for real-time assistance in the interpretation of nCLE on PCLs.

The other difference was the depth of weight layers of VGG. Machicado et al. utilized VGG16, while we adopted VGG19, both of which had similar frameworks except the numbers of weight layers in the architecture: 16 layers in the former and 19 in the latter. Simonyan and Zisserman from the Visual Geometry Group (VGG) of University of Oxford pioneered the VGG algorithm and demonstrated the improved performance of CNN by increasing the weight layers up to 19 layers [22]. The VGG16 processed 134–138 million parameters, and the VGG19 processed 144 million parameters. In their original report, VGG19 had better performance in single test scale than VGG16, but both performed similarly well in multi-test scales.

EUS-guided nCLE evaluation for PCLs provided the unique value of real-time optical pathology and demonstrates clinical utility (sensitivity 87% and specificity 91% for mucinous PCls; 81% and 98% for malignant PCLs) in a recent systematic review investigating 40 studies and 3,641 patients [32]. With a pooled success rate of 88% and adverse event rate of 3%, nCLE will have an increasingly important role in the armamentaria for endoscopists caring for patients with PCLs. Such CAD systems in this study will hopefully provide unique value in this regard.

Our per-frame analysis on the performance of three CNNs among the five subtypes of PCLs revealed different profiles of sensitivity, specificity, and accuracy within the same CNN and across three different methods of designation of ROIs (CNN1-3). Although distinct nCLE features for five subtypes of PCLs have been characterized by experts [10,33], our results suggested that classification performances, even by objective CNN algorithms, were not uniform among five subtypes. In the same topic, Machicado et al. investigated the diagnostic accuracy of 76 nCLE videos by 13 endosonographers (6 experts having > 50 nCLE cases experience) [11]. Table 9 summarizes the comparison between this study and the work by Machicado et al., which implied that non-mucinous PCLs were associated with higher accuracy rates than mucinous PCLs. Our CNN demonstrated superior specificity compared to the state-of-the-art for the classification of mucinous PCLs (IPMN and MCN), with high specificity (94.3% and 92.8%, respectively) but low sensitivity (46% and 45.2%, respectively), which suggests a complimentary role of CNN-enabled CAD systems, especially for clinically suspected mucinous PCLs.

There are several limitations to this study. Firstly, the diagnosis performance still has large room for improvement. Due to the inherent heterogeneity of image contents frame by frame in the fast-moving nCLE videos and the potential selection bias of locating ROIs, mis-classification resulted in suboptimal performance of CNNs, especially in CNN2. Secondly, in per-video analysis, we arbitrarily denoted the highest percentage of subtype as the final subtype of the entire nCLE video. Yet, certain image features in some frames might be more pathognomonic and deserve heavier weight than other frames. Moreover, in real-time practice, we might need to develop an “accumulated frequency score” to denote the final subtyping of the video. Thirdly, our nCLE videos were obtained retrospectively from a single center with limited numbers. Fourthly, we did not include solid pseudopapillary neoplasms, nor did we separate main-ductal type from side-branch type IPMNs. Fifthly, our CNNs might not be applicable to diseases other than PCLs. Lastly and clinically most relevant, the current CAD system was developed and processed off-line on retrospectively collected nCLE video frames, and we have not yet tested prospectively in real-time nCLE examinations. Real-time assistance is urgently needed to facilitate the clinical use of nCLE on PCLs. The speedy computation time of CAD on each frame (0.03–0.07 s) in Table 3 suggests great potential in real-time applications. Future clinical studies incorporating external validation of our CNN algorithms with adequate representation of all five subtypes of PCLs and prospective comparison studies between the CAD system and novice and expert endoscopists are warranted to confirm the performance and clinical utility.

## 5. Conclusions and Future Work

Incidentally detected PCLs are increasing in the asymptomatic general population. The accurate detection of PCLs with malignant potential is a clinical dilemma. We utilized deep learning neural network VGG19 for CAD classification of subtypes of PCLs on EUS-guided nCLE video frames. Our work uniquely compared three different methods of designating ROIs by manual designation, maximal rectangular ROI, and U-Net algorithm designation and validated the use of automatic ROI designation in future CAD systems in nCLE. Our per-frame analysis suggested differential levels of diagnostic accuracy among the five subtypes of PCLs, where non-mucinous PCLs (SCN: 93.11%, NET: 84.31%, and pseudocyst: 98%) had higher diagnostic accuracy than mucinous PCLs (IPMN: 84.43% and MCN: 86.1%). Our CNN demonstrated superior specificity compared to the state-of-the-art for the classification of mucinous PCLs (IPMN and MCN), with high specificity (94.3%, and 92.8%, respectively) but low sensitivity (46% and 45.2%, respectively). Our results will contribute to improve the daily practice of differential diagnosis of PCLs with nCLE. Furthermore, our data revealed a high specificity of nCLE on mucinous PCLs, which carry high clinical importance because mucinous PCLs have higher malignant potential, and early treatment of mucinous PCLs will improve clinical outcomes.

In the future work, we believe machine learning methodologies could solve more complicated problems, such as dissecting the cellular and tissue structures to improve the initial differential diagnosis, monitoring of cellular morphological alterations during surveillance, and exploration of molecular target-labeled fluorescent CLE as molecular imaging of PCLs. In the near future, an “integrative computational model” [13] combining relevant clinical information, nCLE images, radiomics, and next-generation sequencing of pancreatic cystic fluids may collectively provide clinicians with sophisticated diagnosis of PCLs.

## Figures and Tables

**Table 1:** Exemplary views of manually designated ROIs (blue) in test videos.

Subtype	ROI Position	Subtype	ROI Position
IPMN	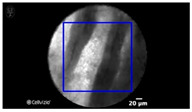	SCN	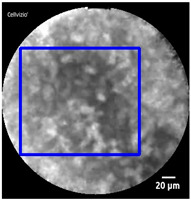
MCN	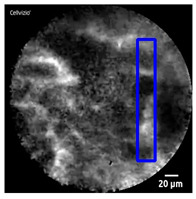	SCN	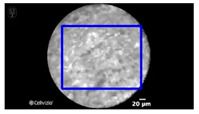
		NET	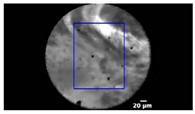
		NET	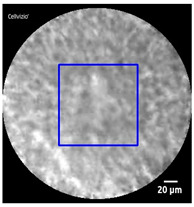
		Pseudocyst	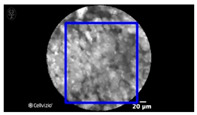

**Table 2:** Exemplary views of U-Net algorithm-designated ROIs (blue) in test videos.

Subtype	ROI Position	Subtype	ROI Position
IPMN	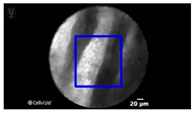	SCN	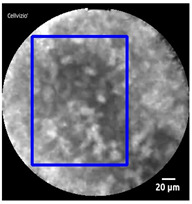
MCN	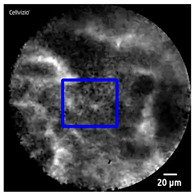	SCN	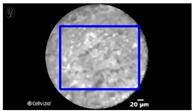
		NET	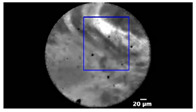
		NET	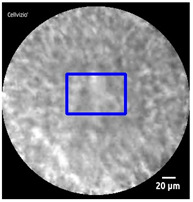
		Pseudocyst	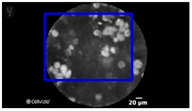

**Table 3:** Test nCLE videos and average processing time by CNN VGG19.

Test Video	Actual Time Video	Average Algorithm Processing Time
IPMN_1	18 s (553 frames)	0.04 s/frame
IPMN_2	31 s (933 frames)	0.04 s/frame
IPMN_3	8 s (210 frames)	0.07 s/frame
IPMN_4	8 s (210 frames)	0.07 s/frame
IPMN_5	26 s (631 frames)	0.05 s/frame
MCN_1	6 s (151 frames)	0.05 s/frame
MCN_2	8 s (210 frames)	0.07 s/frame
MCN_3	49 s (1196 frames)	0.05 s/frame
SCN_1	9 s (285 frames)	0.06 s/frame
SCN_2	123 s (2970 frames)	0.06 s/frame
SCN_3	7 s (182 frames)	0.05 s/frame
SCN_4	10 s (256 frames)	0.05 s/frame
NET_1	8 s (197 frames)	0.07 s/frame
NET_2	8 s (210 frames)	0.07 s/frame
NET_3	28 s (683 frames)	0.04 s/frame
NET_4	58 s (1392 frames)	0.05 s/frame
Pseudocyst_1	18 s (568 frames)	0.03 s/frame
Pseudocyst_2	8 s (210 frames)	0.07 s/frame

**Table 4:** Percentage of subtypes classified by CNN1 with manually selected ROIs in 18 test nCLE videos.

No. of Test Video	Subtype by Ground Truth	Subtype by CNN1 (Largest Percentage)	Correct Subtyping by CNN1	Percentage of Subtypes Classified by CNN1 in Test Videos				
				IPMN	MCN	SCN	NET	Pseudocyst
1	IPMN	IPMN	Yes	32%	30%	5%	31%	2%
2	IPMN	IPMN	Yes	53%	6%	18%	21%	2%
3	IPMN	IPMN	Yes	85%	2%	3%	10%	0%
4	IPMN	IPMN	Yes	48%	26%	0%	0%	26%
5	IPMN	IPMN	Yes	38%	18%	6%	37%	1%
6	MCN	MCN	Yes	26%	31%	25%	1%	17%
7	MCN	MCN	Yes	0%	85%	0%	0%	15%
8	MCN	MCN	Yes	8%	40%	22%	28%	2%
9	SCN	SCN	Yes	0%	0%	95%	15%	0%
10	SCN	SCN	Yes	0%	1%	96%	3%	0%
11	SCN	SCN	Yes	0%	0%	99%	1%	0%
12	SCN	SCN	Yes	0%	0%	99%	1%	0%
13	NET	NET	Yes	2%	0%	0%	98%	0%
14	NET	NET	Yes	22%	7%	10%	59%	2%
15	NET	NET	Yes	8%	3%	3%	86%	0%
16	NET	NET	Yes	18%	16%	1%	66%	0%
17	Pseudocyst	Pseudocyst	Yes	1%	0%	5%	0%	94%
18	Pseudocyst	Pseudocyst	Yes	1%	0%	0%	4%	95%
**Accuracy rate of CNN1 per video**			**100%**					

**Table 5:** Percentage of subtypes classified by CNN2 with maximal rectangular ROIs in 18 test nCLE videos.

No. of Test Video	Subtype by Ground Truth	Subtype by CNN2 (Largest Percentage)	Correct Subtyping by CNN2	Percentage of Subtypes Classified by CNN2 in Test Videos				
				IPMN	MCN	SCN	NET	Pseudocyst
1	IPMN	NET	No	22%	1%	29%	45%	3%
2	IPMN	NET	No	41%	1%	13%	44%	1%
3	IPMN	IPMN	Yes	79%	0%	10%	11%	0%
4	IPMN	SCN	No	9%	6%	40%	35%	10%
5	IPMN	SCN	No	4%	0%	60%	4%	36%
6	MCN	Pseudocyst	No	0%	0%	22%	16%	62%
7	MCN	SCN	No	6%	0%	54%	40%	0%
8	MCN	NET	No	3%	10%	26%	60%	3%
9	SCN	SCN	Yes	0%	0%	93%	1%	6%
10	SCN	SCN	Yes	1%	1%	48%	20%	30%
11	SCN	SCN	Yes	0%	0%	100%	0%	0%
12	SCN	SCN	Yes	0%	0%	83%	1%	16%
13	NET	NET	Yes	5%	0%	21%	54%	20%
14	NET	SCN	No	30%	0%	63%	1%	6%
15	NET	Pseudocyst	No	1%	0%	18%	36%	45%
16	NET	SCN	No	5%	0%	63%	1%	31%
17	Pseudocyst	Pseudocyst	Yes	1%	0%	5%	0%	94%
18	Pseudocyst	IPMN	No	37%	0%	15%	34%	14%
**Accuracy rate of CNN2 per video**			**38.9%**					

**Table 6:** Percentage of subtypes classified by CNN3 with U-Net algorithm-designated ROIs in 18 test nCLE videos.

No. of Test Video	Subtype by Ground Truth	Subtype by CNN3 (Largest Percentage)	Correct Subtyping by CNN3	Percentage of Subtypes Classified by CNN3 in Test Videos				
				IPMN	MCN	SCN	NET	Pseudocyst
1	IPMN	IPMN	Yes	34%	27%	6%	33%	0%
2	IPMN	NET	No	23%	10%	17%	49%	1%
3	IPMN	IPMN	Yes	59%	0%	6%	32%	3%
4	IPMN	NET	No	10%	2%	9%	78%	1%
5	IPMN	IPMN	Yes	76%	1%	8%	5%	10%
6	MCN	SCN	No	0%	0%	36%	33%	31%
7	MCN	NET	No	11%	12%	7%	69%	1%
8	MCN	NET	No	10%	9%	21%	59%	1%
9	SCN	SCN	Yes	0%	0%	89%	4%	7%
10	SCN	SCN	Yes	1%	1%	47%	39%	12%
11	SCN	SCN	Yes	0%	0%	55%	26%	19%
12	SCN	NET	No	1%	0%	9%	67%	23%
13	NET	NET	Yes	16%	11%	20%	43%	10%
14	NET	NET	Yes	33%	7%	14%	44%	2%
15	NET	NET	Yes	34%	1%	2%	61%	2%
16	NET	NET	Yes	25%	4%	23%	34%	14%
17	Pseudocyst	Pseudocyst	Yes	33%	0%	1%	1%	65%
18	Pseudocyst	Pseudocyst	Yes	14%	0%	14%	32%	40%
**Accuracy rate of CNN3 per video**			**66.7%**					

**Table 7:** On a per-frame basis, the performance of three CNNs in relation to different subtypes of PCLs.

	Sensitivity	95% CI	Specificity	95% CI	PPV	95% CI	NPV	95% CI	Accuracy	95% CI
**CNN1_IPMN**	45.95%	44.99–48.91%	94.31%	93.80–94.80%	71.10%	69.09–73.03%	85.64%	85.18–86.09%	83.43%	82.73–84.12%
**CNN1_MCN**	45.22%	42.72–47.73%	92.81%	92.28–93.33%	50.79%	48.53–53.06%	91.17%	90.80–91.53%	86.10%	85.45–86.74%
**CNN1_SCN**	96.29%	95.63–96.88%	91.51%	90.85–92.14%	85.06%	84.09–86.00%	98.00%	97.66–98.30%	93.11%	92.62–93.58%
**CNN1_NET**	73.45%	71.66–75.18%	87.46%	86.74–88.15%	62.93%	61.50–64.33%	91.91%	91.41–92.39%	84.31%	83.62–84.99%
**CNN1_Pseudocyst**	94.22%	92.34–95.75%	98.29%	95.75–98.02%	80.64%	78.23–82.84%	99.56%	99.41–99.67%	98.00%	97.72–98.25%
**Average**	71.03%		92.88%		70.10%		93.26%		88.99%	
**CNN2_IPMN**	28.14%	26.40–29.94%	96.35%	95.92–96.73%	69.68%	66.94–72.25%	81.81%	81.44–82.17%	80.68%	79.93–81.42%
**CNN2_MCN**	7.71%	6.43–9.15%	99.40%	99.22–99.54%	67.80%	60.68–74.18%	86.78%	86.61–86.94%	86.48%	85.82–87.11%
**CNN2_SCN**	56.46%	54.84–58.07%	66.60%	65.51–67.68%	45.91%	44.85–46.98%	75.28%	74.53–76.02%	63.21%	62.30–64.11%
**CNN2_NET**	14.83%	13.45–16.29%	73.68%	72.74–74.61%	14.04%	12.86–15.30%	74.91%	74.52–75.30%	60.46%	59.54–61.37%
**CNN2_Pseudocyst**	72.37%	69.08–75.48%	79.37%	78.58–80.15%	21.00%	20.06–21.97%	97.43%	97.13–97.70%	78.88%	78.11–79.64%
**Average**	35.90%		83.08%		43.69%		83.24%		73.94%	
**CNN3_IPMN**	40.48%	38.56–42.42%	87.39%	86.67–88.09%	48.90%	47.08–50.73%	83.12%	82.65–83.58%	76.62%	75.82–77.40%
**CNN3_MCN**	8.54%	7.20–10.04%	95.97%	95.56–96.36%	25.83%	22.36–29.63%	86.48%	86.29–86.66%	83.65%	82.95–84.34%
**CNN3_SCN**	48.01%	46.39–49.64%	85.97%	85.15–86.75%	63.21%	61.67–64.72%	76.70%	76.12–77.28%	73.28%	72.44–74.10%
**CNN3_NET**	42.99%	41.03–44.96%	61.89%	60.85–62.92%	24.64%	23.67–25.63%	78.93%	78.29–79.56%	57.64%	56.72–58.57%
**CNN3_Pseudocyst**	58.23%	54.67–61.72%	91.78%	91.23–61.72%	34.93%	32.96–36.95%	96.67%	96.39–96.92%	89.42%	88.83–89.99%
**Average**	39.65%		84.60%		39.50%		84.38%		76.12%	

**Table 8:** Comparison of published studies utilizing machine learning algorithms with PCLs.

Author, Year	Neural Network Model	Machine Learning Algorithms	Main Tasks
**Kuwahara, 2019 [17]**	ResNet50	Deep learning on EUS images of IPMN	Diagnosis of IPMN malignancy
**Machicado, 2021 [18]**	VGG16 and mask R-CNN	CNN-CAD algorithms: segmentation-based and holistic-based models	Risk stratification of IPMN, automatic ROI-designation
**Kurita, 2019 [29]**	Basic deep learning neural network	Deep learning of cyst fluid analysis	Differentiate malignant from benign PCLs
**Liang, 2022 [30]**	Radiomics deep learning models	Support vector machines (SVM)	Classification on mucinous vs. non-mucinous PCLs
**Proposed method**	U-Net, VGG19	Deep learning and data preprocessing using LTP feature extraction, Gaussian pyramid, and CLAHE	Classification on five subtypes of PCLs

**Table 9:** Comparison of diagnostic performance on five subtypes of PCLs by objective CNN1 (manually designated ROIs) (this study) and by expert endosonographers ([11]).

	Sensitivity	95% CI	Specificity	95% CI	Accuracy	95% CI
**CNN1_IPMN**	46.0% *	45–48.9%	94.3% *	93.8–94.8%	83.4% *	82.7–84.1%
**Machicado, 2022_IPMN**	84.6%	81.1–87.6%	90.3%	87.5–92.6%	87.6%	85.4–89.5%
**CNN1_MCN**	45.2% *	42.7–47.7%	92.8%	92.3–93.3%	86.1%	85.5–86.7%
**Machicado, 2022_MCN**	64.3%	57.1–70.9%	90.9%	88.8–92.7%	86.0%	83.7–88.1%
**CNN1_SCN**	96.3% *	95.6–96.9%	91.5% *	90.9–92.1%	93.1% *	92.6–93.6%
**Machicado, 2022_SCN**	82.9%	75.1–88.7%	97.5%	96.2–98.3%	95.8%	94.3–96.8%
**CNN1_NET**	73.5% *	71.7–75.2%	87.5% *	86.7–88.2%	84.3% *	83.6–85%
**Machicado, 2022_NET**	86.4%	80.4–90.8%	98.3%	97.2–99%	96.3%	94.9–97.3%
**CNN1_Pseudocyst**	94.2%	92.3–95.8%	98.3%	95.8–98%	98.0%	97.7–98.3%
**Machicado, 2022_Pseudocyst**	92.3%	79.7–97.4%	97.2%	95.9–98%	97.0%	95.7–97.9%

* denotes non-overlapping of 95% CI between data from CNN1 and from Machicado et al.

## Data Availability

Not applicable.

## Supplementary Materials

The following supporting information can be downloaded at: https://www.mdpi.com/article/10.3390/diagnostics13071289/s1, Supplementary Materials contain descriptions of data preprocessing, including Gaussian pyramid, local ternary pattern (LTP) extraction, CLAHE image processing, and sensitivity analysis of parameters in the algorithms. Figure S1: Gaussian pyramid illustration process; Figure S2: LTP ternary code operation; Figure S3: LTP implementation (a) original image. (b) LTP feature extraction image; Figure S4: (a) Original image;\linebreak (b) Processed image with clip limit 1.5 in CLAHE scheme; Figure S5: Graph accuracy of VGG19 model from whole image training; Figure S6: Graph loss of VGG19 model from whole image training; Table S1: The effects of batch size on the accuracy in subtyping of PCLs among 18 test videos during the training of VGG19 model; Table S2: The effect of learning rate on the accuracy in subtyping of PCLs among 18 test videos during the training of VGG19 model; Table S3. The effect of clip limit’s value on the accuracy in subtyping of PCLs among 18 test videos during the training of VGG19 model; Figure~S7: Graph accuracy of U-Net training; Figure S8: Graph loss of U-Net model training; Table~S4: The effect of batch size on the accuracy in subtyping of PCLs among 18 test videos during the training of U-Net model; Table S5: The effect of learning rate on the accuracy in subtyping of PCLs among 18 test videos during the training of U-Net model.
