# Supplementary material for: Deep-Learning-Enabled Computer-Aided Diagnosis in the Classification of Pancreatic Cystic Lesions on Confocal Laser Endomicroscopy
Source: Diagnostics (Basel). 2023 Mar 29;13(7):1289. doi: 10.3390/diagnostics13071289 (PMC10093377; doi:10.3390/diagnostics13071289)
Supplement: Supplementary file 1 [file diagnostics-13-01289-s001.zip › diagnostics-2231316-supplementary.pdf]

Supplementary Materials

S1. Data preprocessing

S1.1. Gaussian pyramid

One of the best methods for obtaining multiresolution features in an image is to use an image pyramid. Image pyramids are usually divided into two types: Gaussian pyramids and Laplacian pyramids. A Gaussian pyramid is a down sampled multiscale image representation. To obtain the down-scaled image, the Gaussian pyramid is performed in two steps. First, we convolution the original image with the Gaussian kernel (k). The Gaussian kernel is illustrated in Equation (1). Second, to obtain the down-scaled image, the even-numbered rows and columns are removed.

$$k = \frac{1}{16}\begin{bmatrix} 1 & 4 & 6 & 4 & 1 \\ 6 & 16 & 24 & 16 & 4 \\ 6 & 24 & 36 & 24 & 6 \\ 4 & 16 & 24 & 16 & 4 \\ 1 & 4 & 6 & 4 & 1 \end{bmatrix} \quad (1)$$

Figure S1 illustrates a Gaussian pyramid with four levels. The size decreases as the layer number increases. An X by Y image becomes an X/2 by Y/2 image, with the area gradually reduced to one-fourth of its original size.

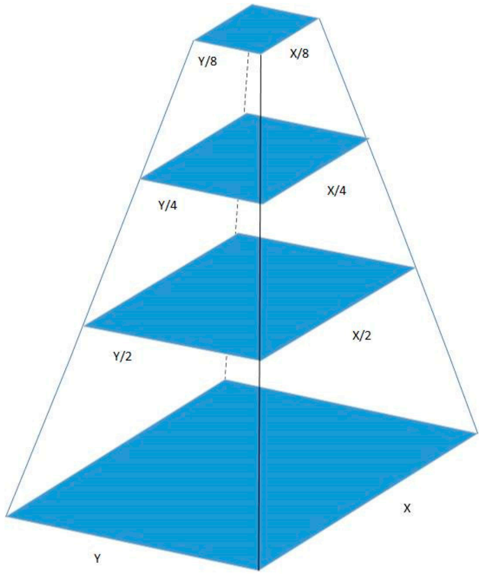

Figure S1. Gaussian pyramid illustration process

S1.2. Local ternary pattern (LTP) extraction

The local ternary pattern (LTP) feature of the pancreatic cystic images was extracted and used as the training image patterns in the VGG19 model. LTP is well-known for content-based image retrieval and is robust to process the image noises [1]. LTP is a 3-valued ternary code. Equation (2) is used to calculate the ternary code for each pixel in an image by comparing the value of the central pixel to the values of the neighboring pixels.

$$\mathrm{LTP} = \begin{cases} 1 & V \geq i + t \\ 0 & |V - i| < t \\ -1 & V \leq (i - t) \end{cases}, \tag{2}$$

where *V*, *i*, and *t* represent the grayscale values of neighboring pixels, the center pixel, and the threshold value, respectively. Figure S2 illustrates an example of calculating the LTP ternary code with *t* = 5. It is also illustrating the original pixel values and the corresponding LTP ternary code. The LTP ternary code can also be divided into two distinct channels, LTP upper and LTP lower codes. Both the corresponding LTP upper and lower codes for each pixel are 8 bits long and can be reformed to two grayscale values of the pixel. In addition to the existing training image, two more grayscale images generated by both LTP codes can be obtained. In our method, we also include these two LTP images in our training dataset.

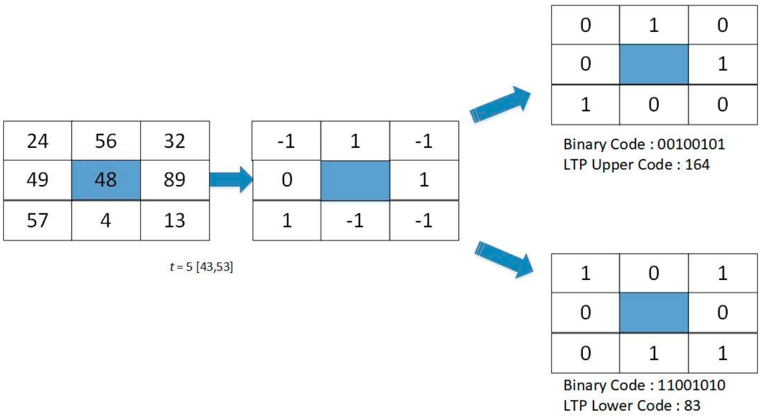

Figure S2. LTP ternary code operation

We use LTP feature extraction only on IPMN. Figure S3 shows the implementation before (S3a) and after (S3b) the LTP feature extraction.

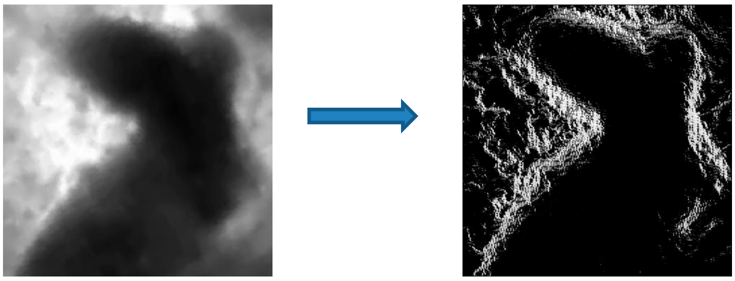

Figure S3. LTP implementation: (a) original image. (b) LTP feature extraction image.

S1.3. CLAHE image processing

Adaptive histogram equalization (AHE) is a common image preprocessing method to improve image contrast. It is different from general histogram equalization because it calculates a specified area and then uses the computed value to redistribute the image brightness so that the image contrast can be adopted. This method is suitable for improving the local contrast of the image and enhancing the edges in the image to obtain more details. However, AHE has a problem. While improving the local contrast and enhancing the edges in the image, it also enlarges the noise of the image. To overcome this issue, Yadav et al. proposed the Contrast Limited Adaptive Histogram Equalization (CLAHE) method [2]. Unlike AHE, CLAHE limits the contrast in each small area by limiting the contrast of AHE. CLAHE in medical images can nonlinearly modify the image pixel values to maximize the contrast of all pixels in the image. Figures S3a and S3b show an example of CLAHE being applied to a given test image with the clip limit set to 1.5.

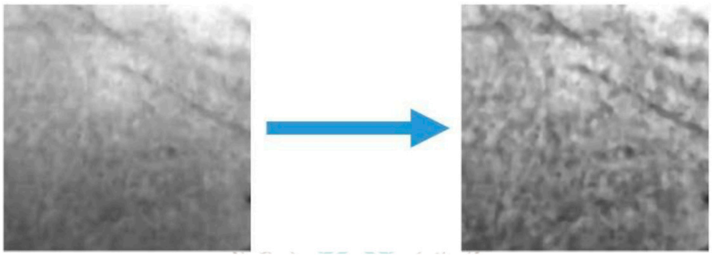

(a) (b)

Figure S4. (a) Original image; (b) Processed image with clip limit 1.5 in CLAHE scheme.

S2 Sensitivity tests on parameters of our proposed algorithm

S2.1. Training parameters for VGG19 architecture:

We use the sparse categorial cross-entropy (SCCE) as our loss function for VGG19 because we have 5 subtypes of PCLs as our outputs and we want to get the percentage of likelihood from each of the subtypes.

For the epoch number, we observe the model accuracy and model loss during the VGG19 network training process. As we can see from Figs. S5 and S6, the accuracy and loss curves have obvious fluctuations and start to converge after the 55th iteration. We choose 100 epochs because the model accuracy and loss are stable without fluctuations.

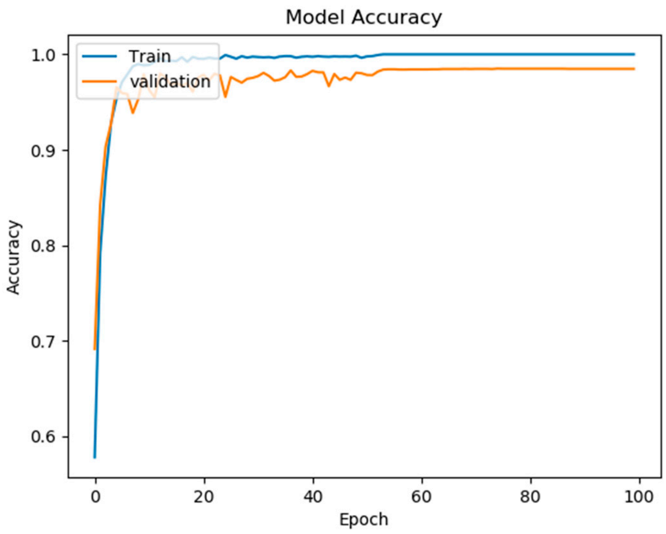

Figure S5：Graph accuracy of VGG19 model from whole image training

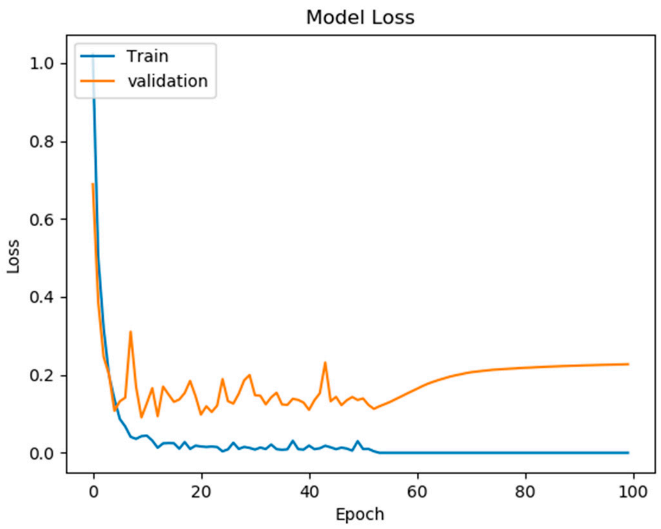

Figure S6：Graph loss of VGG19 model from whole image training

For the batch size, we try several numbers of batch sizes of 32, 16, 8, and 4, respectively. From the experimental results, we observe the sensitivity of the batch size to the accuracy. Table S1 shows the numbers of correctly subtyped videos corresponding to the four batch sizes. As we can observe from the table, the best accuracy is obtained by using the batch size 8.

Table S1. The effects of batch size on the accuracy in subtyping of PCLs among 18 test videos during the training of VGG19 model.

No.	Batch size	Correctly subtyped videos
1.	32	6
2.	16	9
3.	8	12
4.	4	8

For the learning rate, we test four different values to find the best learning rate. Table S2 shows the correlation between the learning rate and the numbers of correctly classified videos. According to this table, the best learning rate for the VGG19 model is 0.0001.

Table S2. The effect of learning rate on the accuracy in subtyping of PCLs among 18 test videos during the training of VGG19 model.

No.	Learning rate	Correctly subtyped videos
1.	0.00001	9
2.	0.0001	12
3.	0.001	11
4.	0.01	11

For the clip limit parameter used in CLAHE, three clip limit values are tested with the value of 1.0, 1.5, and 2.0, respectively. Table S3 shows that the best clip limit value is 1.5.

Table S3. The effect of clip limit's value on the accuracy in subtyping of PCLs among 18 test videos during the training of VGG19 model.

No.	Clip limit's value	Correctly subtyped videos
1.	1.0	11
2.	1.5	12
3.	2.0	11

S2.2. Parameters for U-Net:

We use the binary cross-entropy as our loss function in U-Net network because we only have two classes (features and background) as our segmentation result.

For epoch number, we observe the model accuracy and model loss during the U-Net network training process. As we can see from the Figs. S7 and S8, the accuracy and loss curves have obvious fluctuations and start to converge after the 70th iteration. Therefore, we choose 100 epochs because the model accuracy and loss are stable without fluctuations.

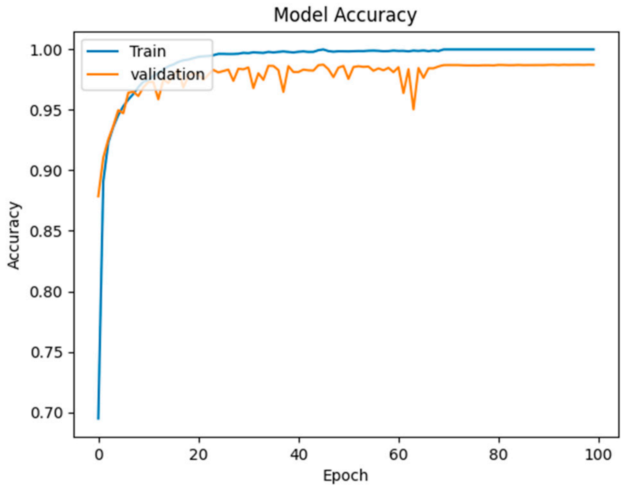

Figure S7：Graph accuracy of U-Net training

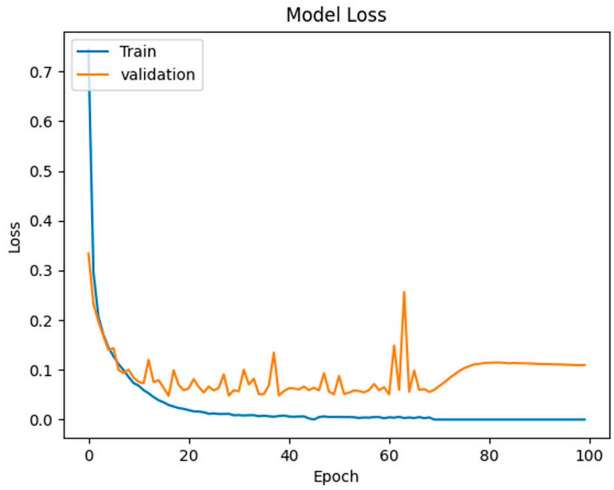

Figure S8：Graph loss of U-Net model training

For the batch size, we try several batch size's numbers of 32, 16, 8, and 4, respectively. From the experimental results, we observe the sensitivity of the batch size to the accuracy. Table S4 shows the numbers of correctly subtyped videos corresponding to the four batch sizes. As we can observe from the table, the best accuracy is obtained by using the batch size 8.

Table S4. The effect of batch size on the accuracy in subtyping of PCLs among 18 test videos during the training of U-Net model.

No.	Batch size	Correctly subtyped video
1.	32	12

2.	16	10
3.	8	11
4.	4	11

For the learning rate, we test four different values to find the best learning rate. Table S5 shows the correlation between the learning rate and the numbers of correctly classified videos. According to this table, the best learning rate for the U-Net model is 0.001.

Table S5. The effect of learning rate on the accuracy in subtyping of PCLs among 18 test videos during the training of U-Net model.

No.	Learning rate	Correctly classified videos
1.	0.00001	8
2.	0.0001	10
3.	0.001	12
4.	0.01	8

References

1. Reddy, K.S.K., V.V.; Reddy, B.E., Face recognition based on texture features using local ternary patterns. International Journal of Image, Graphics and Signal Processing (IJIGSP), 2015. 7(10): p. 37-46.

2. Yadav, G.M., S.; Agarwal, A., Contrast limited adaptive histogram equalization based enhancement for real time video system, in 2014 International Conference on Advances in Computing, Communications and Informatics (ICACCI). 2014: New Delhi, India. p. 2392-2397.
